# Seeking evidence to support efforts to increase use of antenatal care: a cross-sectional study in two states of Nigeria

**DOI:** 10.1186/s12884-014-0380-4

**Published:** 2014-11-20

**Authors:** Khalid Omer, Nshadi John Afi, Moh’d Chadi Baba, Maijiddah Adamu, Sani Abubakar Malami, Angela Oyo-Ita, Anne Cockcroft, Neil Andersson

**Affiliations:** CIET Trust, 71 Oxford Road, Saxonwold, Johannesburg, 2196 South Africa; Ministry of Health, Bauchi State Government, Bauchi, Nigeria; Ministry of Health, Cross River State Government, Calabar, Nigeria; CIET Trust Botswana, PO Box 1240, Gaborone, Botswana; CIET-PRAM, Department of Family Medicine, McGill University, Montreal, Canada

**Keywords:** Antenatal care, Government, Determinants, Cross-sectional, Multivariate, Nigeria

## Abstract

**Background:**

Antenatal care (ANC) attendance is a strong predictor of maternal outcomes. In Nigeria, government health planners at state level and below have limited access to population-based estimates of ANC coverage and factors associated with its use. A mixed methods study examined factors associated with the use of government ANC services in two states of Nigeria, and shared the findings with stakeholders.

**Methods:**

A quantitative household survey in Bauchi and Cross River states of Nigeria collected data from women aged 15–49 years on ANC use during their last completed pregnancy and potentially associated factors including socio-economic conditions, exposure to domestic violence and local availability of services. Bivariate and multivariate analysis examined associations with having at least four government ANC visits. We collected qualitative data from 180 focus groups of women who discussed the survey findings and recommended solutions. We shared the findings with state, Local Government Authority, and community stakeholders to support evidence-based planning.

**Results:**

40% of 7870 women in Bauchi and 46% of 7759 in Cross River had at least four government ANC visits. Women's education, urban residence, information from heath workers, help from family members, and household owning motorized transport were associated with ANC use in both states. Additional factors for women in Cross River included age above 18 years, being married or cohabiting, being less poor (having enough food during the last week), not experiencing intimate partner violence during the last year, and education of the household head. Factors for women in Bauchi were presence of government ANC services within their community and more than two previous pregnancies. Focus groups cited costly, poor quality, and inaccessible government services, and uncooperative partners as reasons for not attending ANC. Government and other stakeholders planned evidence-based interventions to increase ANC uptake.

**Conclusion:**

Use of ANC services remains low in both states. The factors related to use of ANC services are consistent with those reported previously. Efforts to increase uptake of ANC should focus particularly on poor and uneducated women. Local solutions generated by discussion of the evidence with stakeholders could be more effective and sustainable than externally driven interventions.

## Background

Antenatal care (ANC) is an important component of maternal health services. Studies in India and Nigeria have documented an association between maternal morbidity and mortality and non-use of ANC [1-3]. ANC is an opportunity to provide women with information and services that can help to reduce the risk of morbidity and mortality for mother and child [4,5]. This is particularly relevant in Nigeria, where maternal mortality is estimated at 608/100,000 live births in 2008 (uncertainty range 372–927) [6], among the highest in the world.

Based on the evidence on effectiveness of care components in routine ANC [7-9] and a randomized trial of a new approach to promotion of safe pregnancy [10] the World Health Organisation recommends at least four ANC visits for pregnant women without complications. The Federal Ministry of Health in Nigeria recommends four ANC visits as part of its national strategic health development plan 2010–15 [11]. However, the use of ANC services in Nigeria is low. The 2008 Nigeria Demographic and Health Survey [12] indicated only 45% of women aged 15–49 years received four ANC visits from a skilled provider during their last pregnancy, with a big urban (69%) rural (34%) differential. The 2011 Multiple Indicator Cluster Survey [13] conducted in Nigeria reported 50% coverage with four ANC visits, suggesting little progress since 2008.

Socio-economic and service delivery factors including accessibility are well recognized determinants of the use of ANC [14,15]. A systematic review of 28 studies identified women's and their husbands’ education, economic status, parity, place of residence and accessibility to health services as key determinants of use of ANC services [16]. There is some published literature from Nigeria documenting such factors. However, most of this either comes from institutional data [3] or small studies in specific areas [17-22]. Some authors have analysed data from national surveys to examine factors related to use of ANC in Nigeria [23-25], but published data on determinants of use of ANC services at state or Local Government Authority (LGA) level are lacking.

The health care system in Nigeria provides autonomy to the states and Local Government Authorities for planning and delivery of health services. Government run facilities are often the only formal health care option, especially for poor people in rural areas. State and Local Government Authority planners mostly rely on information from national surveys such as Nigerian Demographic and Health Survey and Multiple Indicator Cluster Survey to assess and monitor progress of their health indices. However, the sample size for the individual states and Local Government Authorities limits use of the national evidence at these levels. A national health management information system is theoretically in place [26]. However, the system has so far proved inefficient and ineffective for collection, collation and use of data [27]. Relying heavily on facility based data, this information system also excludes those who do not access the facilities.

This paper analyses data from a cross-sectional survey conducted as part of a social audit process under the auspices of the Nigerian Evidence Based Health System Initiative in 2009 [28] in two Nigerian states, Bauchi and Cross River. Bauchi is situated in the north east zone of the country with a predominantly Muslim population and polygamy is common. Cross River is in the south-eastern zone, the main religion as Christianity, and families tend to be more nuclear. Using a sizeable representative sample from all the Local Government Authorities in the two states, the social audit process aimed to provide robust population-based evidence to support health planning at state and Local Government Authority levels [29,30]. This paper addresses the research questions: what proportion of women use government ANC services in Bauchi and Cross River States? What are the factors associated with use of these services in each state? The paper also describes how the state-specific findings were shared with relevant stakeholders in the two states to support evidence-based planning of service improvements.

## Methods

Between September and October 2009 as part of a state wide social audit we undertook a cross-sectional household survey on maternal outcomes and associated factors in Bauchi and Cross River states of Nigeria [2]. The stratified random, cluster sample was drawn from the sample frame of enumeration areas from the 2006 census. It comprised 90 clusters in each state: 10 sites in each of three randomly selected focus Local Government Authorities and 60 among the remaining Local Government authorities to give state-level representation.

Design groups led by the Ministry of Health in each state developed a household questionnaire, seeking information about demographics and socio-economic status of the household, and a questionnaire for women aged 15–49 years, asking about their own socio-economic status and their knowledge and attitudes about maternal health and care. The questionnaire for women asked those who reported a completed pregnancy in the previous three years about their use and experience of antenatal care during their last pregnancy. An additional key informant questionnaire sought information about access to health services in each cluster. Field teams pre-tested the questionnaires, translated into the local language, in non-sample communities in each state.

Training of local fieldworkers covered questionnaire administration and recording of responses, and comprised both classroom and field practice sessions. Each field team comprised two male and eight female interviewers with one male and one female supervisor. Radiating from a random central starting point in each cluster, interviewers visited contiguous households until they reached the target number of women (approximately 100), with no further sub-sampling within the cluster.

Three months after the household survey, selected members of the same field teams returned to each of the same clusters to share and discuss the findings about the use of ANC services with focus groups of women. In each cluster the field team of two women conducted a focus group discussion with 10–12 women from among those who had participated in the earlier household survey, based on their availability and willingness. The teams conducted 180 focus groups in total, involving around 1,800 women. The facilitators used a guide to feedback key findings from the survey and to invite discussion within the group. The facilitator invited the group participants to give their views about why women do not attend government ANC services and to suggest possible actions to increase use of these services. The other team member took detailed notes of the discussion. The discussions were in the local language of the area, but the team members prepared their reports of the discussions in English.

### Analysis

Different operators entered all the data twice with validation using Epi Info [31] to minimise keystroke errors. Analysis relied on CIETmap open source software [32]. We weighted all estimates proportional to the population in each state, including rural and urban characteristics, and adjusted for over-sampling in the focus Local Government Authorities. We did not undertake a combined analysis of the two states, since they were not intended to be representative of a larger entity (such as the nation). Rather we analysed the data for the two states separately, since an explicit aim was to share state-specific findings to support planning at state level and below.

The main outcome was four or more ANC visits to a government health facility in the last pregnancy. Factors examined for their association with the outcome included location of residence (urban or rural), whether the household or individual woman had enough food during the week prior to the survey (as an indicator of absolute poverty), household owning motorised transport, education of the household head and the woman, the woman's exposure to domestic and intimate partner violence, access to health information, number of previous pregnancies, woman receiving help for her routine heavy work at home during pregnancy, and access to health facilities whether within or outside the community (see Table 1). We defined “more education” as junior secondary or above in Cross River and any formal education in Bauchi. In order to produce state specific information about factors associated with use of ANC, we examined the associations in bivariate and then multivariate analysis in each state separately. For the multivariate analysis we used the Mantel Haenszel procedure [33] adjusted for clustering [34] and stepped-down from an initial saturated model including all the variables found significant in the bivariate analysis until we reached a final model of factors all significantly associated with the outcome. We describe associations using the Odds ratio (OR) with the cluster adjusted 95% confidence interval (caCI).

**Table 1:** **Characteristics of women aged 15–49 years reporting a pregnancy during the last 3 years**

	**% (n/N)**	
**Characteristics**	**Cross river**	**Bauchi**
Had at least four ANC visits to a govt. health facility	46 (3259/6943)	40 (3012/7441)
From urban household	30 (2569/7568)	18 (1243/7861)
*Age (at time of survey)*		
15-18 years	9 (6377562)	16 (1175/7845)
19-35 years	82 (6267/7562)	77 (6108/7845)
36-49 years	9 (658/7562)	7 (562/7845)
*Education level*		
No education	7 (515/7524)	48 (3646/7808)
Some primary	23 (1760/7524	13 (1050/7808)
Some junior secondary	15 (1145/7524)	2 (120/7808)
Some senior secondary	37 (2724/7524)	6 (434/7808)
Diploma or higher	18 (1380/7524)	1 (94/7808)
Informal (Arabic/Islamic)	-	30 (2464/7808)
Married or cohabiting	81 (6150/7558)	97 (7644/7851)
Had enough food for themselves during the last week	82 (6142/7552)	90 (7063/7836)
Gainfully employed	59 (4428/7558)	48 (3741/7800)
Received information on maternal health issues from a health worker	71 (5319/7542)	42 (3526/7755)
Involved in decision on where to go for ANC (alone or with husband)	25 (1932/7545)	0.5 (41/7812)
Considered a government health facility as the nearest one for maternal care	84 (6166/7333)	96 (7358/7666)
Did not experience intimate partner violence during last year	80 (5992/7482)	96 (7484/7808)
*Number of previous pregnancies*		
No previous pregnancy	24 (1717/7341)	14 (1158/7742)
One previous pregnancy	21 (1552/7341)	18 (1372/7742)
Two previous pregnancies	17 (1237/7341)	16 (1251/7742)
Three or more previous pregnancies	38 (2835/7341)	51 (3961/7742)
Received help with work from a family member during pregnancy	78 (5654/7335)	68 (5428/7846)
*Education level of household head*		
No education	11 (809/7424)	30 (2030/7360)
Some primary	21 (1528/7424)	11 (842/7360)
Some junior secondary	8 (565/7424)	2 (206/7360)
Some senior secondary	35 (2667/7424)	11 (818/7360)
Diploma or higher	25 (1855/7424)	8 (565/7360)
Informal (Arabic/Islamic)	-	37 (2899/7360)
From household having enough food during last week	79 (5919/7525)	83 (6177/7451)
From household with own motorized transport	40 (2921/7493)	39 (2894/7454)
From community with a govt. health facility providing ANC services	71 (3808/5304)	27 (2404/7460)
From community with good access road	37 (2104/5216)	31 (2495/7225)
From community with a village development committee	77 (4039/5304)	35 (2482/7460)

Three of the authors (KO, NJA and MCB) undertook a thematic analysis of the translated focus group reports, to identify emerging themes about reasons for non-attendance for ANC. They continued reading through reports until no new themes emerged, and extracted relevant quotes to illustrate the different themes. We used the qualitative findings from the focus groups to give context to and help to explain the quantitative findings from the household survey in the two states.

### Using the findings

During the period from April 2010 till March 2011 we shared the study findings with stakeholders at State, Local Government Authority and community levels. We designed a score card showing values of key indicators for each Local Government Authority with the state average value for comparison. A video docudrama portrayed key messages based on the findings. We conducted workshops, small group and individual meetings with ministries, departments and non-government organisations at state and Local Government Authority level to share the findings and plan actions to increase the use and quality of ANC services. The meetings were timed to allow proposed actions to be included in the budget allocations for 2010–2012.

Field teams conducted structured discussions around the docudrama with community leaders and groups of men and women in order to generate local solutions. The state Ministry of Health in Cross River and the state Primary Health Care Development Agency in Bauchi, with support from the state Ministry of Health, used the evidence to implement a model intervention of pregnancy surveillance in three randomly selected focus Local Government Authorities [35] in each state to improve women's access to ANC. Under this intervention trained female and male health workers and community activists periodically visit all the households in a defined catchment area assigned to them. They use structured evidence based guides to interact with pregnant women, their spouses, and family members to generate an enabling environment for improving care for pregnant women and their access to ANC. They aim to visit each pregnant woman at least four times during the pregnancy.

### Ethics

The Ministry of Health in each state gave formal ethical approval for the study. The trained field teams sought consent for the survey from leaders in each sample community. In each household the interviewers sought verbal consent from the household head, and from each individual respondent. Interviewers did not record any names or personal identity information and were trained not to proceed with any interview unless they could do so without being overheard.

## Results

**Figure 1:**
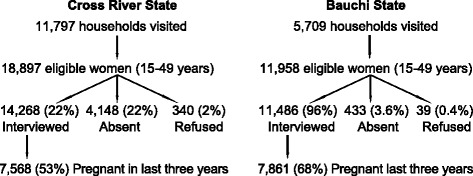
**Women in the study.**

Figure 1 shows the sample study population in the two states. About one fifth (22% - 4148/18897) of eligible women (aged 15–49 years) in Cross River were not available in the visited households, mostly because they were out at work. Women more often work outside the household in Cross River than in Bauchi. A higher proportion of interviewed women in Bauchi than in Cross River reported a pregnancy during the last three years.

Table 1 shows characteristics of women reporting a pregnancy during the three years preceding the survey. A total of 46% (3259/6943) in Cross River and 40% (3012/7441) in Bauchi reported having at least four antenatal care checkups at a government health facility. Compared with Bauchi, more women in Cross River had formal or junior secondary education, received information on health care and were involved in making decision on where to go for antenatal care. Also the proportion of women from communities having a health facility providing antenatal care or a village development committee was higher in Cross River than in Bauchi.

Table 2 shows the bivariate associations of potential factors associated with the outcome of having at least four government ANC visits in each state. Significant associations are marked in bold. In both states, women were more likely to have four ANC visits if: they were from urban areas, aged 18 years or above, more educated, from a household with a more educated household head, less poor (having enough food during the last week), had received information on health during pregnancy and child birth, were from households with motorised transport, and were from communities with a government health facility providing antenatal care. Additional factors for Cross River included: being married or co-habiting, not experiencing intimate partner violence during last year or last pregnancy, naming a government health facility as their nearest source of antenatal care, and coming from a less poor household (having enough food during last week). For Bauchi additional factors included: being gainfully employed, having more than two previous pregnancies, being from a community with a good access road and from a community with an active village development committee.

**Table 2:** **Bivariate associations between potential factors and having four ANC visits among women aged 15–49 years with a pregnancy in the last three years**

	**Cross river**			**Bauchi**		
	**% (n/N) with 4 ANC visits**		**OR (95% caCI)**	**% (n/N) with 4 ANC visits**		**OR (95% caCI)**
	**With factor**	**Without factor**		**With factor**	**Without factor**	
From urban household	54 (1255/2311)	43 (2004/4632)	**1.56** **(1.18 - 2.05)**	63 (747/1192)	36 (2265/6249)	**2.95 (2.08 - 4.19)**
Current age >18 years	48 (3048/6342)	35 (210/596)	**1.70 (1.36 - 2.12)**	41 (2618/6303)	34 (389/1123)	**1.34 (1.15 - 1.57)**
With some formal education	48 (3070/6434)	36 (172/472)	**1.59 (1.31 - 1.94)**	63 (1019/1620)	34 (1978/5787)	**3.27 (2.60 - 4.10)**
With junior secondary or higher education	51 (2424/4792)	39 (816/2109)	**1.62 (1.37 - 1.92)**	67 (405/603)	38 (2583/6791)	**3.33 (2.33 - 4.76)**
Married or co-habiting	49 (2756/5684)	40 (499/1249)	**1.41 (1.22-1.65)**	41 (2933/7235)	38 (74/197)	1.13 (0.80-1.60)
Had enough food for themselves in last week	49 (2737/5621)	39 (514/1308)	**1.47 (1.28-1.69)**	41 (2761/6674)	32 (237/743)	**1.51 (1.16-1.95)**
Gainfully employed	48 (1942/4063)	46 (1311/2870)	1.09 (0.98-1.21)	45 (1588/3539)	37 (1405/3843)	**1.41 (1.21-1.64)**
Received Info on pregnancy issues from a health worker	52 (2533/4877)	35 (715/2042)	**2.01 (1.75 - 2.30)**	53 (1783/3384)	30 (1196/3963)	**2.58 (2.14-3.10)**
Involved in decision on where to go for ANC (alone or with husband)	46 (809/1773)	47 (2443/5152)	0.93 (0.82-1.06)	56 (22/39)	40 (2975/7356)	1.91 (0.91-4.00)
Considered a govt facility as nearest for ANC	49 (2764/5660)	37 (399/1073)	**1.61 (1.37-1.89)**	41 (2845/6973)	39 (110/279)	1.06 (0.70-1.59)
Did not experience IPV in the last year	49 (2674/5502)	41 (558/1369)	**1.37 (1.23-1.54)**	40 (2846/7083)	49 (152/308)	0.69 (0.45-1.06)
With more than 2 previous pregnancies	47 (1253/2674)	47 (1993/4244)	1.00 (0.91 - 1.09)	44 (1636/3763)	37 (1336/3574)	**1.29 (1.16-1.43)**
Not beaten during last pregnancy	48 (2926/6113)	39 (290/742)	**1.43 (1.24-1.66)**	41 (2892/7127)	48 (86/179)	0.74 (0.48 - 1.13)
Had help with work from a family member during last pregnancy	50 (2647/5335)	38 (610/1602)	**1.60 (1.39-1.85)**	44 (2282/5137)	32 (727/2298)	**1.73 (1.45-2.06)**
From household with head having junior secondary or higher education	51 (2313/4563)	40 (888/2250)	**1.58 (1.35-1.84)**	60 (914/1515)	35 (1894/5466)	**2.87 (2.28-3.60)**
From household with enough food in last week	49 (2640/5426)	41 (598/1478)	**1.39 (1.21-1.61)**	41 (2390/5846)	38 (467/1223)	1.12 (0.94-1.33)
From household with own motorized transport	53 (1407/2661)	43 (1814/4215)	**1.49 (1.33-1.66)**	50 (1361/2717)	34 (1501/4360)	**1.91 (1.62-2.26)**
From community with good access road	52 (981/1900)	47 (1356/2889)	1.21 (0.89-1.63)	53 (1256/2380)	34 (1529/4469)	**2.15 (1.48-3.12)**
From community with a govt facility providing ANC	52 (1825/3516)	43 (579/1357)	**1.45 (1.04-2.02)**	59 (1354/2303)	32 (1520/4766)	**3.05 (2.22-4.19)**
From community with a village development committee	49 (1805/3714)	52 (599/1159)	0.88 (0.58-1.34)	53 (1235/2352)	35 (1639/4717)	**2.08 (1.48-2.90)**

Note: “With factor” column shows the proportion who had 4 ANC visits among those with the factor (eg from an urban community); “Without factor” column shows the proportion who had 4 ANC visits among those without the factor (eg from a rural community).

“caCl” means cluster adjusted confidence interval.

Significant associations are marked in bold.

Table 3 shows the final multivariate models. In both states, a woman was more likely to have four ANC visits during her last pregnancy if she was more educated, if she had help from family members during the pregnancy, if she received information on pregnancy issues from health workers, if she lived in an urban community, and if her household owned some form of motorized transport. In Cross River, less-poor women (having enough food in the last week), those who did not experience physical intimate partner violence during the year preceding survey, and those who named a government health facility as their nearest source of ANC (rather than a non-government facility) were more likely to have four government ANC visits. In Bauchi women with more than two previous pregnancies and residing in communities with a government health facility providing ANC services were more likely to have four government ANC visits.

**Table 3:** **Multivariate analysis of factors associated with having 4 ANC visits in last pregnancy, among women aged 15–49 years with a pregnancy in the last three years**

**Factors**	**Cross river**	**Bauchi**
	**OR (95% caCI)**	**OR (95% caCI)**
Received information on pregnancy issues from a health worker	1.75 (1.51-2.02)	2.06 (1.73-2.45)
From urban household	1.54 (1.21-1.96)	1.58 (1.18-2.13 )
Had help during pregnancy from family members	1.37 (1.19-1.59)	1.27 (1.06-1.51)
From household with own motorized transport	1.32 (1.17-1.49)	1.22 (1.06-1.40)
From household where head had junior secondary education or above	1.16 (1.01-1.34)	-
Current age >18 years	1.49 (1.20-1.84)	-
Considered govt. health facility as the nearest facility for ANC services	1.48 (1.27-1.72)	-
Did not experience IPV in the last year	1.24 (1.11-1.38)	-
Married or co-habiting	1.24 (1.04-1.47)	-
From household with enough food during last week	1.20 (1.05-1.37)	-
With junior secondary or higher education	1.26 (1.06-1.48)	-
With some formal education	-	1.85 (1.51-2.26 )
Had more than two previous pregnancies	-	1.28(1.15-1.44)
From community with a government health facility providing ANC	-	1.95 (1.50-2.53)

### Views from the focus groups

Many of the women participating in focus groups cited problems with services delivery as the reason they could not or did not attend for ANC. High cost was a frequent complaint.

*“One of my friends went for antenatal care and they told her to pay N5,000.00 [USD 31]. She came back home crying that she does not have money”. (Focus group participant, Cross River).*

Tying in with the higher use of ANC in urban areas, in those households with their own motorised transport, and in communities with a facility providing ANC, many focus groups cited poor access to health facilities as an important barrier particularly in rural communities.

*“The major problem is that we don’t have a health facility in this community and we don’t have money to transport ourselves” (Focus group participant, Bauchi).*

Even when they could reach a health facility, women complained that the services were poor. Health workers failed to attend, treated women badly, especially if they were poor, and demanded unofficial payments. Equipment and supplies were inadequate. This supports the quantitative finding that women from the poorest households were less likely to attend for ANC.

Reflecting the quantitative finding that more educated women were more likely to attend for ANC, another theme from the focus groups was to blame women themselves for not attending because they were uneducated, “careless”, afraid of injections, or did not see the point or need of attending for ANC.

*“Our mothers never went to the hospital for delivery, why should we?” (Focus group participant, Cross River).*

In Bauchi, many focus groups noted that husbands did not allow their wives to attend for antenatal care, sometimes because this would reflect badly on them.

*“If he allows her to go it will be said that he is being over ridden by his wife, so he will be seen as a very weak man that has no power over his wife”. (Focus group participant, Bauchi).*

Some groups also explained that a woman could have problems with her in-laws if she asked to attend for ANC.

*“When a woman stays with her inlaws the moment she starts talking about antenatal care the inlaws will complain that she wants to spend the son’s money by going to the hospital, so she forgets about it so that they don’t paint her black”. (Focus group participant, Bauchi)*

Suggestions for how to increase the use of government ANC services largely mirrored the complaints. They wanted free care, accessible care, and good behaviour from health workers.

*“Government should make available free medical care/drugs to all pregnant women during antenatal care. They should also supply sufficient drugs”. (Focus group participant, Bauchi).*

*“Health facilities should be brought nearer to our communities and government should employ qualified health workers to work there. Nurses should always check the blood pressure and urine of pregnant women whenever they go for ANC” (Focus group participant, Bauchi).*

*“Health workers should be friendly and polite to their patients and treat them all equally. They should always be at their duty post” (Focus group participant, Cross river).*

Focus groups in both the states called for creating awareness about the importance of ANC visits, especially among men, and young women in their first pregnancy.

*“Our husbands should be sensitized on the importance of antenatal check-ups so that they will help us and encourage us to go”. (Focus group participant, Bauchi)*

### Use of the findings

Following dissemination of study findings, the Ministries of Health in both the states through Primary Health Care departments in the Local Government Authorities, deployed more health workers, including Community Health Extension Workers (CHEWs), midwives and nurses, especially in rural areas. In Bauchi, the Local Government Authorities made financial allocations to improve staff supervision and monitoring, and provide equipment and medicines to the facilities.

Also in both states, community groups of men, women and youth in the three focus Local Government Authorities who viewed the docudrama implemented local arrangements, for example to help women to avoid heavy work during pregnancy, to get adequate rest, and to attend ANC. They took local actions to create awareness about danger signs during pregnancy and child birth. They organized counselling through traditional and religious leaders to address domestic violence. In Bauchi, several community groups organized a local referral system for obstetric emergencies, including providing emergency transport and financial support. They established links with their nearest referral hospitals, to ensure they are ready to receive and deal with these emergencies.

Home visits for pregnancy surveillance in both states have encouraged male involvement, so that men support their wives during pregnancy and encourage them to attend ANC. The home visitors have informed women about what they should expect when they visit the health facility for ANC. Anecdotal evidence from the facilities suggests that women are now making requests for blood pressure measurement and urine testing during ANC visits.

## Discussion

Our study revealed that less than half of pregnant women in Bauchi and Cross River states had the recommended four ANC visits; this is consistent with findings from other national and sub-national studies in Nigeria [12,13,24]. The 2008 Nigeria Demographic and Health Survey (national sample size 33,385 women aged 15–59 years) reported that 45% of women (15–49 years) nationally who gave birth in the last five years had four ANC visits [12]. The 2011 Multiple Indicator Cluster Survey reported that among women with a live birth in the last two years, 29% in Bauchi (of 455) and 60% in Cross River (of 203) had at least four ANC visits in their last pregnancy [13].

Use of government ANC services in both states was higher in urban areas, in households owning motorized transport, among more educated women, among women who had help from family members during pregnancy, and among those receiving information on pregnancy related issues from a health worker. Similar associations have been reported in other studies [12,20-24,36-43]. Women in urban areas are likely to be better informed with better access and more choice of services. Availability of motorized transport increases family access to health facilities. Poor access to ANC services was a frequent complaint in focus group discussions, especially in Bauchi. The association between receiving information from a health worker on pregnancy related issues and use of ANC services may be because the information creates awareness of the importance of ANC. Or it could be because women who attended ANC were given information about pregnancy issues at these visits. Education of household heads and support from family members may well be a proxy for their better understanding about care needed by women during pregnancy.

Some factors influencing the use of government ANC services differed between the two states. In Cross River, married or cohabiting women, those above the age of eighteen, those from less poor households, and those not experiencing intimate partner violence during the last 12 months were more likely to have four or more government ANC visits. These associations are consistent with findings from other studies [2,22,23,35,38,43,44]. Cross River is predominantly a Christian society; young women who are not married or cohabiting may face problems attending ANC. These factors were not associated with attending for ANC in Bauchi. In this predominantly Muslim society, there are strong disincentives for unmarried women to report a pregnancy.

In Bauchi, women with two or more previous pregnancies were more likely to have attended for ANC during their last pregnancy. This contrasts with a study in Bangladesh, which reported that women were more likely to attend for ANC during a first pregnancy, perhaps due to perceived higher risks in this pregnancy [43]. A possible explanation for our finding comes from the suggestion in some focus groups that women requesting antenatal care may be labelled adversely by their in-laws. In a polygamous society like Bauchi, this could discourage women from demanding and using ANC for their initial pregnancies, fearing that it may disadvantage them in relation to other wives, or lead their husband to take another wife.

Including all Local Government Authorities in the sample was helpful and allowed local planners to assess the use of government ANC services in each Local Government Authority. The quantitative analysis of factors related to use of ANC services, together with the qualitative findings form the focus groups, has provided useful pointers towards local interventions to improve the situation. The careful design of communication tools and methods helped to share the evidence with different audiences, and the timing fitted into the budget and planning cycle. Analysis of the findings from a recent further survey in the two states will help to evaluate the effects of the various interventions introduced as a result of the evidence-based discussions with government and other stakeholders in each state. This approach of collecting and communicating local population based evidence could be used in other states to support evidence-based health planning.

### Limitations

As with all cross-sectional studies, we can only document associations and we do not know the direction of potential causality. In Cross River more than a fifth of the eligible women were not available at the time of the survey, mostly because they were out at work. This may have introduced a selection bias in the estimate of the use of government ANC services. It is less likely to have affected associations between different factors and the use of services. And, indeed, the analysis did not find a significant association between gainful employment and the use of ANC services.

## Conclusion

There is a continuing low level of use of government ANC services in both states. The factors we found related to use of ANC services are consistent with those reported previously. Efforts to increase use of ANC need to focus particularly on poor, uneducated women in rural areas. Local solutions generated by discussion of the evidence take into account the local context and could be more effective and sustainable than externally driven interventions.

## Abbreviations

ANC: Antenatal care
CIET: Community Information Empowerment and Transparency
caCI: Cluster adjusted confidence interval
OR: Odds Ratio
UNICEF: United Nations Children Fund
